# Dr. Charles Bigelow Braman

**Published:** 1914-10

**Authors:** 


Dr. Charles Bigelow Braman, Buffalo, 1898, Chief Pharmacist of the Clifton Springs Sanitarium, died July 4, aged 53.

